# Antimicrobial Resistance Gene Profiles in Integron-Positive and Integron-Negative Third-Generation Cephalosporin-Resistant *E. coli* from Human and Animal Sources

**DOI:** 10.3390/antibiotics15050427

**Published:** 2026-04-24

**Authors:** Tin Ho, Liseth Salinas, Gabriel Trueba, Heather K. Amato, Nikolina Walas, Mihir Pandya, Timothy Johnson, Jay Graham

**Affiliations:** 1 Environmental Health Sciences Division, University of California, 2121 Berkeley Way West, Berkeley, CA 94720, USA; tin@berkeley.edu (T.H.); nikolina.walas@ucsf.edu (N.W.); mihir_pandya@berkeley.edu (M.P.); 2 Instituto de Microbiología, Colegio de Ciencias Biológicas y Ambientales, Universidad San Francisco de Quito, Quito 170901, Ecuador; lsalinast1@usfq.edu.ec (L.S.); gtrueba@usfq.edu.ec (G.T.); 3 Department of Global, Environmental, and Occupational Health, School of Public Health, University of Maryland, 4200 Valley Drive, College Park, MD 20740, USA; hkamato@umd.edu; 4 Department of Veterinary and Biomedical Sciences, University of Minnesota, 1971 Commonwealth Avenue, Saint Paul, MN 55108, USA; tjj@umn.edu

**Keywords:** *E. coli*, ESBL, integron, One Health, whole genome sequencing, antimicrobial resistance

## Abstract

**Background/Objectives**: Integrons are genetic platforms that allow bacteria to acquire antimicrobial resistance (AMR) genes, making them a focal point for many AMR studies and surveillance programs. This study investigated how the prevalence of integrons (*intI* and *attI* genes) in third-generation cephalosporin-resistant *E. coli* (3GCR-Ec) varied across three different sources (i.e., healthy children, domestic animals and urinary tract infections). The study aimed to determine how different classes of AMR genes vary among 3GCR-Ec with integrons present versus those where integrons are absent. **Methods**: We analyzed 3GCR-Ec isolates collected from semirural parishes of Eastern Quito, Ecuador, that included: (1) 3GCR-Ec from healthy children (*n* = 946), (2) 3GCR-Ec from domestic animal species (*n* = 673), and 3GCR-Ec from patients with urinary tract infections (UTIs) (*n* = 138). Genomic analyses were performed for all 1757 sequences to determine how the presence and absence of integrons was associated with AMR gene carriage. **Results**: Among the total sequences of 3GCR-Ec evaluated across all datasets, nearly one-third (31%) were integron-negative. 3GCR-Ec from UTI patients, however, had a higher percentage containing integrons (79%). Across all sets of 3GCR-EC, integron-positive isolates carried an average of 10.3 (±3.0 SD) AMR genes versus 4.8 (±2.5 SD) AMR genes in integron-negative isolates. This study found that between 21% to 33% of 3GCR-Ec across the three different sources lacked integrons but maintained the ability to carry diverse classes of AMR genes, including beta-lactams, aminoglycosides, tetracyclines, and multidrug resistance mechanisms (e.g., general-purpose efflux pumps). **Conclusions**: While integrons were associated with greater AMR genes on average, the study highlights that solely relying on integrons for tracking drug-resistant bacteria misses a substantive portion of AMR that is present in integron-negative strains.

## 1. Introduction

Integrons are genetic elements that serve as platforms for genetic exchange. They have core features that include an integrase gene (*intI*), a recombination site (*attI*) and a promoter (Pc). These genetic elements allow integrons to capture and express external genes as part of gene cassettes recombined into the *attI* site [1,2]. Integrons enable bacterial evolution through the addition, subtraction, collection, and recombination of open reading frames in cassettes [3]. Complete integrons typically consist of several consecutively ordered genetic components, beginning with an integrase gene (*intI*), followed by a variable array of gene cassettes, each flanked by a recombination site (*attC*) [4].

The ability of integrons to acquire and mobilize antimicrobial resistance (AMR) genes is considered a major contributor to the emergence of multidrug-resistant bacteria [5,6]. Consequently, integrons have been widely adopted as a proxy for anthropogenic pollution and commonly used as an indicator in AMR surveillance [7,8]. They are considered important markers of AMR in microbial communities [9], and are also used to estimate antimicrobial concentrations in environmental reservoirs such as wastewater [10]. Researchers have shown the undeniable role that integrons play in the evolution of AMR and have highlighted the need to better understand the mechanistic behavior of integrons, as well as the implications that more multidrug-resistant bacteria pose to humans [11,12]. Others, however, have found that AMR genes are often present in non-integron-containing isolates [13,14].

Orthogonally, third-generation cephalosporin-resistant *Escherichia coli* (3GCR-Ec) have emerged as a critical concern to public health. Third-generation cephalosporins, such as ceftriaxone, cefotaxime, and ceftazidime, are frequently prescribed due to their established safety profile and clinical utility. The widespread and sometimes inappropriate use of these broad-spectrum antibiotics in clinical settings has been a significant driver of bacterial evolution and the proliferation of resistance [15], some of which commonly circulates in communities [16]. 3GCR-Ec isolates commonly harbor AMR genes, such as extended spectrum beta-lactamases (ESBLs), rendering them resistant to numerous currently available antibiotics [17,18]. This can pose a therapeutic challenge for patients suffering from life-threatening conditions such as sepsis and meningitis. Additionally, research has highlighted that high-risk *E. coli* strains are commonly transmitted between humans and animals, complicating control strategies [19]. In 2021, an estimated 1.14 million deaths were attributable to antibiotic-resistant bacterial infections; most occurred in low- and middle-income countries [20]. With limited treatment options, cephalosporin-resistant and ESBL-producing bacterial infections result in longer and more costly hospital stays, increased severity of illness, and increased risk of mortality.

In a previous study [21], we found extensive overlap between isolates from children, dogs, and chickens in 222 households in peri-urban communities of Quito, Ecuador, sampled across five timepoints. Among them, ST10, ST155, ST117, ST2847, ST162, ST38, and ST354 were dominant. Moreover, ESBL genes (*bla*_CTX-M-15_ *bla*_CTX-M-27_, and *bla*_CTX-M-3_) were of significantly higher prevalence in human isolates compared to both dog and chicken isolates. To better understand the role of integrons in clinically relevant bacterial populations, we analyzed the prevalence of integron carriage and AMR genes across three distinct sets of 3GCR-Ec previously collected from healthy children, domestic animals, and patients with urinary tract infections (UTIs) in semirural parishes of Eastern Quito, Ecuador.

## 2. Results

### 2.1. Integron Prevalence in 3GCR-Ec

A total of 946 3GCR-Ec isolates were obtained from healthy children living in a semi-rural region of Ecuador where commercial and domestic livestock production is common [21]. Out of 946 isolates, 631 (66.7%) were integron-positive. The integron-positive group harbored a total of 6350 resistance genes, corresponding to an average of 10.1 (±2.9 SD) AMR genes per isolate. Conversely, 315 isolates (33.3%) were integron-negative and collectively harbored 1446 resistance genes, with an average of 4.6 (±2.3 SD) AMR genes per isolate (Table 1).

We analyzed 673 isolates collected from domestic animals (e.g., dogs, chickens, pigs, ducks, cows, and geese) within the same communities as the healthy children (Table 1). Among the animal isolates, 467 were found to be integron-positive (69.4%) and averaged 10.6 (±3.2 SD) AMR genes per isolate. The remaining 206 isolates were integron-negative (30.6%) and had an average of 5.1 (±2.8 SD) AMR genes per isolate.

Integron carriage was highest in the 138 clinical 3GCR-Ec isolates from patients with a urinary tract infection (UTI) (Table 1). In this group of isolates, 109 contained integrons (79.0%). These isolates averaged 10.3 (±2.3 SD) AMR genes per isolate. The 29 integron-negative isolates (21.0%) possessed an average of 5.5 (±2.6 SD) AMR genes per isolate. Overall, the integron-negative isolates consistently had fewer AMR genes than the integron-positive group.

### 2.2. AMR Genes in Integron-Positive and Integron-Negative Isolates

**Figure 1:**
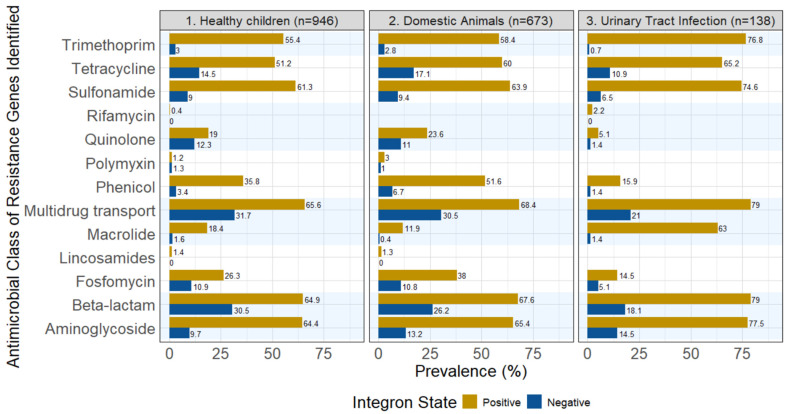
Prevalence of AMR genes aggregated by antimicrobial classes across the three sampling sources. All isolates were 3GCR-Ec. Multidrug transport is a summary class of efflux pump genes such as *mdf*, *mdt*, *nor*, and *oqx*. Supplementary Tables S1–S3 contain the data presented in the figure.

Figure 1 summarizes the number of AMR genes present in integron-positive versus integron-negative isolates. These genes conferred resistance to major drug classes, including first-line treatments (e.g., beta-lactamases and quinolones) and critically important antimicrobials such as polymyxin, alongside multidrug efflux pump genes (e.g., *mdf*, *mdt*, *nor*, and *oqx*). We grouped the resistance genes into 13 classes of AMR; nearly all classes were found in our isolates, whether they were integron-positive or integron-negative, with the exceptions of rifamycin and lincosamides, which were found with very low prevalences in integron-positive isolates, and absent in integron-negative isolates. Notably, the prevalence of polymyxin resistance was slightly higher in the integron-negative group in the healthy children dataset.

The diversity of AMR genes in 3GCR-Ec from domestic animals (Figure 1) was similar to those found in 3GCR-Ec from healthy children, with comparable proportions across most classes.

For the 3GCR-Ec from UTI patients, resistance genes for 11 antimicrobial classes, out of 13, were detected; polymyxin and lincosamides were not detected. Of the classes detected, the dominant classes were trimethoprim, tetracycline, sulfonamide, multidrug transport (efflux pumps), beta-lactamase and aminoglycoside, although they tended to have a higher prevalence than the isolates from healthy children and domestic animals. Similar to the other 3GCR-Ec, AMR carriage was still found in integron-negative isolates in most classes, except for rifamycin; their prevalence compared to integron-positive isolates was also significantly lower. On the other hand, resistance to macrolides was much higher in the clinical UTI isolates than in the isolates from healthy children and domestic animals. For example, the integron-positive group had a macrolide resistance gene prevalence of 63% in 3GCR-Ec from UTI patients versus 18% in 3GCR-Ec from children and 11% in 3GCR-Ec from domestic animals. For the integron-negative isolates, the prevalence ranged from 0.4% in 3GCR-Ec from domestic animals to 1.6% in 3GCR-Ec from children. AMR genes were not identified for the lincosamide and polymyxin classes and quinolone and rifamycin AMR genes were found at a very low prevalence (See Supplemental Table S3 for data).

### 2.3. Beta-Lactamase Genes in Integron-Positive and Integron-Negative Isolates

**Figure 2:**
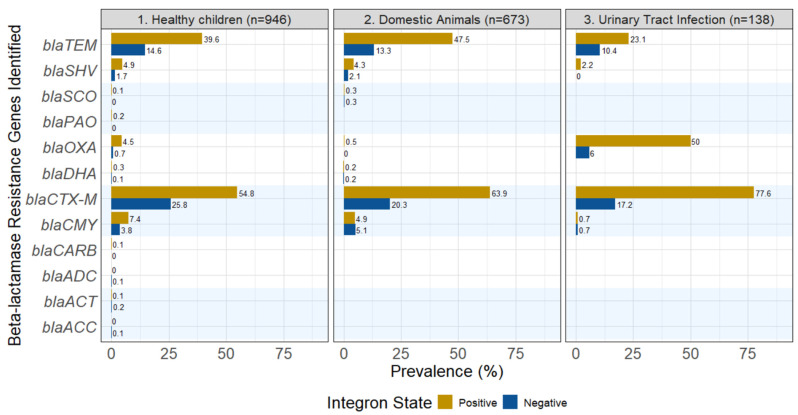
Prevalence of specific beta-lactamase genes for integron-positive and integron-negative 3GCR-Ec from healthy children, domestic animals and patients with urinary tract infections.

The prevalence of different beta-lactamase genes varied greatly across the 3GCR-Ec isolates from healthy children. The dominant beta-lactamase gene type was *bla*_CTX-M_, showing up in 54.8% of the integron-positive isolates from healthy children. The same *bla*_CTX-M_ genes were still present in integron-negative isolates, but at lower levels (25.8%). Other frequent beta-lactamase genes included *bla*_TEM_, *bla*_CMY_ and *bla*_SHV_. Their prevalences were higher in the integron-positive isolates from the children, but even in the absence of integrons, they were still present: 14.6% for *bla*_TEM_, 3.8% for *bla*_CMY,_ and 1.7% for *bla*_SHV_. An additional eight beta-lactamase genes were found in a small proportion of isolates from healthy children (Figure 2).

Among the isolates from domestic animals, the prevalences of various beta-lactamase genes were similar to those observed in children. Again, *bla*_CTX-M_ was the most dominant resistance gene (63.9% for integron-positive isolates and 20.3% for integron-negative isolates), with *bla*_TEM_, *bla*_CMY_ and *bla*_SHV_ following as second, third and fourth in prevalence. Integron-positive isolates had higher prevalences, and integron-negative isolates had nearly one-third to one-half of the beta-lactamase genes as those found in integron-positive isolates.

In the 3GCR-Ec from UTI patients, *bla*_CTX-M_ was again the most dominant resistance gene (77.6% for integron-positive isolates and 17.2% for integron-negative isolates). In contrast to the other isolates described above, integron-positive 3GCR-Ec from UTIs commonly had *bla*_OXA_ (50.0% in integron-positive isolates versus 6.0% in integron-negative isolates). The *bla*_TEM_ gene was the third most common beta-lactamase for integron-positive isolates, but second among integron-negative isolates (Figure 2). The beta-lactamase genes, *bla*_SHV_ and *bla*_CMY_, were also identified in 3GCR-Ec from UTI patients, but at lower prevalences.

### 2.4. Sequence-Type Distribution in Integron-Positive and Integron-Negative Isolates

**Figure 3:**
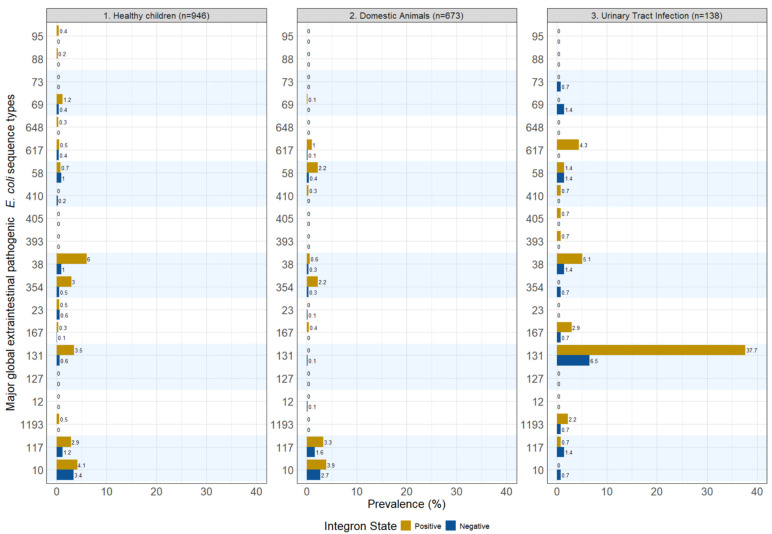
Presence and absence of integrons among the top 20 ExPEC Sequence Types for 3GCR-Ec isolated from healthy children, domestic animals and patients with urinary tract infections [22]. Note: The prevalence in this figure is a percentage of the total within the corresponding sample type. They do not add to 100%, as many more sequence types were present beyond the top 20 ExPEC lineages reported in this figure.

We compared integron carriage by known extraintestinal pathogenic *E. coli* sequence types (STs) (Figure 3). Specifically, we presented integron carriage among the top twenty global extraintestinal pathogenic *E. coli* sequence types based on a systematic review of the most prevalent extraintestinal pathogenic *Escherichia coli* (ExPEC) lineages identified in studies of extraintestinal infections [22]. Overall, the top twenty STs had a low prevalence across our sampled populations. ST131 was the most notable ST, commanding a total of 44.2% from our UTI patient cohort; 37.7% were integron-positive and 6.5% were integron-negative. Among healthy children, ST131 had 3.5% and 0.6% integron-positive and integron-negative prevalence, respectively. ST131 was essentially zero among the domestic animal isolates. ST38 had a similar distribution pattern as that of ST131. Across all of the isolates in each dataset, integron-positive ST38 was present in 5.1% of the UTI patient samples, 6% of the healthy children samples, and 0.6% among the domestic animal samples; integron-negative ST38 prevalence dropped to 1.4%, 1% and 0.3%, respectively, across these datasets. Two other sequence types, ST10 and ST117, were more prevalent among healthy children and domestic animals, but less so among patients with UTIs. Integron-positive ST 10 had prevalences of 4.1% and 3.4% in patients with UTIs and domestic animals, respectively, but was negative in the healthy children set. Some STs were seen at higher prevalence in their integron-negative state, including: ST69, ST73, ST354 and ST117 for patients with UTIs. Many of the top 20 global ExPEC lineages were absent in the three datasets. There were a total of 156 unique STs found among the healthy children, 144 STs among the domestic animals, and 34 STs among patients with UTIs.

## 3. Discussion

*Escherichia coli* is notorious for horizontal gene transfer, an important pathogen in local infections, and the principal cause of UTIs and bacterial bloodstream infections [23]. Integrons are common genetic motifs frequently found in many bacteria, including *E. coli*. The prevalence of integrons in bacterial populations of South America has not been widely studied. In 2024, however, Solis et al. [24] reported finding integrons in almost one third of the *E. coli* isolates they collected from Andean countries, which included Venezuela, Peru, Ecuador, Colombia and Bolivia. The researchers highlighted that the prevalence of integrons within *E. coli* populations found in Ecuador was the highest, at nearly 40%. Our study also adds to this literature, and we found integron prevalence within 3GCR-Ec isolates averaging 69% across the three sources studied.

This present analysis of 3GCR-Ec isolates supports the notion that integrons are indeed important genetic elements associated with a higher burden of AMR genes in resistant *E. coli*. The data also highlight that integron-negative isolates constitute a significant source of AMR genes, containing an average of 4.8 (±2.5 SD) AMR genes. This value is lower than the average for those of integron-positive isolates, which contained an average of 10.3 (±3.0 SD) AMR genes, but not an insignificant number.

Prior studies have established that integron prevalence is source-dependent, suggesting a distinct function for integrons in host–pathogen co-evolutionary dynamics [25]. Furthermore, specific sequence types of *E. coli* lineages are well-known for their global dissemination and association with antimicrobial resistance [26]. Our findings from the diverse 3GCR-*Escherichia coli* samples collected in the semirural parishes near Quito, Ecuador, support the notion that integron-positive *E. coli* are indeed important strains associated with a higher burden of resistance genes. However, our data also highlight that integron-negative isolates constituted a significant source of antimicrobial resistance genes. While integron-positive isolates averaged a high number of resistance genes (approximately 10 per isolate), the integron-negative isolates were far from benign. The integron-negative isolates carried an average of nearly five AMR genes per isolate. Thus, diverse resistance genes in integron-negative isolates can drive multidrug resistance, complicating clinical treatment [27,28].

We observed a high diversity of AMR gene classes in both integron-positive and integron-negative strains across all three populations of 3GCR-Ec. The widespread occurrence of multidrug efflux pump genes (e.g., *mdf*, *mdt*, *nor*, and *oqx*) was also a concern. The detection of fosfomycin resistance is also of medical significance, although it is rarely prescribed in Ecuador for UTIs [29,30].

Integron-negative *E. coli* isolates accounted for approximately 30% of the total AMR genes detected in the beta-lactamase and multidrug resistance (efflux pump) categories. For example, integron-negative isolates were responsible for around 15% of the total AMR genes detected for both tetracycline and quinolone resistance; this is a small fraction, but not a completely insignificant contribution.

There were strong similarities between the 3GCR-Ec from healthy children and the domestic animals in terms of the prevalence of integrons, AMR genes, and more specifically, the prevalence of beta-lactamase genes. This finding highlights that human and domestic animal *E. coli* populations may be frequently exchanged due to interactions between humans, their animals, and their environments [31,32].

There were some cases where antimicrobial resistance to specific classes seemed unrelated to whether the isolate was integron-positive or negative. For instance, resistance to polymyxin, while low in overall prevalence (approximately 1%), was detected with similar frequency in both integron-positive and integron-negative isolates. In contrast, quinolone resistance in integron-positive isolates was nearly half that of integron-negative isolates (19% vs. 12% in 3GCR-Ec from children and 23% vs. 11% in 3GCR-Ec from domestic animals). These two classes of antimicrobials are also non-trivial, as both quinolones and polymyxins are classified as Highest Priority Critically Important Antimicrobials (HPCIA) by the World Health Organization [33]; thus, the presence of those resistance genes is concerning.

While integron presence was associated with more beta-lactamase genes on average, they were not essential for beta-lactamase gene carriage [34]. After removing all integron-positive isolates, there would still remain a prevalence of *bla*_CTX-M_ in 25.8% of the isolates from healthy children, and 17.2% of the isolates from patients with UTIs. The case for *bla*_TEM_, while lower, would still account for 14.6% and 10.4% of 3GCR-Ec from children and UTI patients, respectively. These numbers, while lower, are not low enough to be medically ignored for their contribution to human health. Interestingly, although *bla*_CTX-M_ genes are transferred between bacteria by mobile genetic elements, like transposable elements [35], reports have detected these genes within the integron’s variable region (the cassette) [36,37].

While there were specific differences in the AMR classes and prevalence of integrons found between the isolates in healthy humans versus domestic animals, on the aggregate, we found them to be very similar. This is likely due to frequent exchange of bacterial populations and their resistance genes between humans and animals living in proximity.

In terms of integron carriage by sequence types known to cause disease globally, we found that integron-positive isolates were more prevalent across most STs. This finding aligns with the distribution of integron carriage across various resistance genes discussed above. The most notable sequence type was ST131 in the UTI patient dataset. ST131 is the most frequent ST among the top 20 global ExPEC lineages as reported by a systematic review by Manges et al. [22]. In our UTI patient cohort, 37.7% and 6.5% of the isolates are attributed to the integron-positive and integron-negative isolates, respectively, totaling 44.2%.

Several key pandemic sequence types—specifically ST69, ST73, ST354 and ST10—were present, but were exclusively integron-negative isolates in our UTI patient dataset. We note that ST69 is the second-highest among the top 20 ExPEC STs, and no integron-positive isolates were found among our UTI patients. This study showed that integrons are often present in many multidrug-resistant lineages like ST131, but absent in many pandemic lineages.

There were several limitations to this study. While healthy children and domestic animal isolates were collected at the same community concurrently, UTI isolates were collected during a different time period. The temporal gap between the collection of UTI isolates (2014–2015) and subsequent samples (2019–2021) is a limitation. Given the rapid evolution of AMR, the older isolates may reflect a baseline resistance profile that has since shifted due to changing antibiotic stewardship practices or the emergence of new clones in the intervening five years. Additionally, we did not determine the genomic location of AMR genes nor whether these genes were present within integron-associated gene cassettes. The relative positioning of AMR genes with respect to integrons warrants further investigation, as analyses would provide valuable mechanistic insights into the role of integrons in shaping AMR gene repertoires. However, the primary objective of this study was to assess how the presence or absence of integrons within an isolate influences AMR gene prevalence across different populations of 3GCR-Ec. The study showed that there was discriminatory power with the use of integrons, but that important AMR genes were still found in integron-negative isolates. Future studies may also benefit by further elucidating whether certain farm animals, or pets, may confer the same or different AMR risks.

In this study, we analyzed sequence data from three sets of 3GCR-Ec derived from healthy children, domestic animals and patients with a UTI to better understand how integrons are associated with antimicrobial resistance gene carriage. Studies have shown that *E. coli* lineages that cause UTIs, versus commensal lineages, may experience higher selective pressure as they are exposed more often to antibiotic treatments [38]. The carriage of integrons in the UTI isolates was likely associated with this selective pressure. Our findings indicate that while integrons may be associated with a higher prevalence of AMR genes, important resistance determinants are also common in integron-negative isolates. This highlights the need for future analyses that integrate integron carriage with bacterial lineage and AMR gene profiles to better understand the mechanisms driving AMR dissemination in commensal and pathogenic *E. coli* populations.

## 4. Materials and Methods

### 4.1. 3GCR-Ec from Healthy Children

A total of 1699 fecal samples from 600 children were collected from seven semirural parishes east of Quito, Ecuador, as part of an existing study conducted by researchers at the Universidad San Francisco de Quito (USFQ), Ecuador [21]. A total of 376 households were involved, with repeated sample collection spanning over 3 years (2019–2021). 3GCR and ESBL-producing *E. coli* from the samples were determined phenotypically: Samples were cultured on MacConkey agar (Difco, Sparks, MA, USA) with 2 mg/L of ceftriaxone and incubated for 18 h at 37 °C [39]. Up to 2 ceftriaxone-resistant isolates phenotypically matching *E. coli* were selected from each plate. The identity of presumptive *E. coli* colonies was confirmed by culture on Chromocult coliform agar (Merck KGaA, Darmstadt, Germany), at 37 °C for 24 h, through its β-D-glucuronidase activity, followed by the multi-substrate API RapiD-20E identification system (bioMérieux, Marcy l’Etoile, France) using a cut-off of 95%. Confirmed *E. coli* were preserved at −80 °C in trypticase soy broth medium (Difco, Sparks, MA, USA) containing 20% glycerol. 3GCR-EC isolates for each sample were thawed and regrown on MacConkey agar at 37 °C for 18–24 h for evaluation of antibiotic susceptibility by the disk diffusion method (Kirby–Bauer test) on Mueller–Hinton agar (Difco, Sparks, MA, USA). Presumptive *E. coli* colonies were confirmed on Chromocult coliform agar (Merck, Darmstadt, Germany). DNA was extracted from the isolates using Wizard Genomic DNA Purification (Promega, Madison, WI, USA) kits and DNEasy Blood & Tissue Kits (QIAGEN, Hilden, Germany) according to the manufacturer’s protocol. Confirmed 3GCR-*E. coli* isolates were sequenced at the University of Minnesota Genomic Center using Illumina MiSeq or NovaSeq with Nextera XT libraries (San Diego, CA, USA).

### 4.2. 3GCR-Ec from Domestic Animals

The study included 1871 domestic animal fecal samples from the same households in semirural parishes of Ecuador as described above. The sampling area, approximately 320 km^2^, includes both commercial food animal operations and small-scale “backyard” animal production. Samples were cultured and DNA extraction was done as described in Section 4.1 above. Animals sampled included chickens, pigs, guinea pigs, cattle, sheep and goats. For a detailed breakdown of their composition, please see [21].

### 4.3. 3GCR-Ec from Patients with a Urinary Tract Infection

A total of 149 ceftriaxone-resistant *E. coli* isolates were collected from nine clinics east of Quito, Ecuador, serving the same general community as the above healthy children and domestic animals. As previously described [40], samples were taken from patients with a UTI over a 12-month period between May 2014 and May 2015. Samples were sent to the USFQ team for bacterial culture and analysis. Each bacterial isolate was cultured on Chromocult agar (Merck, Darmstadt, Germany). *E. coli* were identified through β-D-glucuronidase activity and preserved at −80 °C in Brain Heart Infusion (BHI) medium, containing 20% glycerol. On each plate, a single bacterial colony was selected and inoculated into 3 mL of trypticase soy broth (TSB) at 37 °C for 18–24 h. DNA from the bacterial isolates was extracted with the QIAGEN DNeasy Blood & Tissue kit using the manufacturer’s instructions. QC was done using the NanoDrop One (Thermo Scientific, Madison, WI, USA). Whole genome sequencing was performed using Illumina NextSeq 2000 (San Diego, CA, USA).

### 4.4. Whole Genome Sequencing and Bioinformatics Analyses

De novo assemblies were performed using SPAdes v3.15 and contigs were joined into a scaffold with a fixed gap size of 100 bp [41]. Integron carriage was identified using Integron Finder 2.0.2, using linear topology, a distance threshold of 4000 bp, a calin threshold of 2, and a minimum and maximum attC size of 40 and 200 bp, respectively [2,42]. An isolate was classified as integron-positive if it contained either a complete or partial integron, and negative if no integron was detected. The rationale for this method was that some researchers might perform integron detection without regard to whether the integron was complete or partial; thus, we matched on the lowest common denominator. AMR genes were determined by ABRicate 1.0.1 [43] using the ResFinder database version 4 which covers 13 classes of AMR genes [7,44] using default parameters. Data analysis was performed using RStudio 2025.09.2 [45].

To determine the sequence type (ST) for each isolate, species identity was first confirmed via ribosomal multi-locus sequence typing (rMLST) v0.1.8 [46,47]. Confirmed *E. coli* isolates were then characterized using traditional MLST to assign specific STs (Seeman MLST v 2.19.0) [48,49]. Integron carriage for each isolate was ascertained. The prevalence of specific lineages was calculated by dividing the number of isolates of a given ST and integron status by the total number of isolates within each respective dataset (healthy children, domestic animals, and patients with urinary tract infections).

## 5. Conclusions

Integrons play a significant role in the evolution of *E. coli* and the dissemination of antimicrobial resistance genes, underscoring the need for continued study. This analysis of three distinct *E. coli* populations from healthy children, domestic animals and UTI patients demonstrated that a substantial proportion of *E. coli* isolates (31%) were integron-negative yet collectively harbored a significant burden of AMR (an average of 4.8 AMR genes per isolate). While integrons are pivotal in the evolution and dissemination of resistance in *E. coli*, the findings highlight the limitations of using them as a sole proxy for AMR. To more accurately track AMR across the One Health interface, surveillance programs should adopt a broader genomic strategy that accounts for diverse mobilization elements relevant to clinical outcomes.

## Figures and Tables

**Table 1:** Counts and percentages of integron carriage and antimicrobial resistance genes for third-generation cephalosporin-resistant *E. coli* from healthy children, domestic animals and urinary tract infections. *p*-values were calculated using the Mann–Whitney test for the integron-positive vs. negative isolates. (Note. SD = standard deviation).

		Integron-Positive		Integron-Negative		
Source of Isolates	Number of Isolates	Number (%) of Isolates	Average Number of Resistance Genes ± SD	Number (%) of Isolates	Average Number of Resistance Genes ± SD	*p*-Value
3GCR-Ec from healthy children	946	631 (66.7%)	10.1 ± 2.9	315 (33.3%)	4.6 ± 2.3	<0.0001
3GCR-Ec from domestic animals	673	467 (69.4%)	10.6 ± 3.2	206 (30.6%)	5.1 ± 2.8	<0.0001
3GCR-Ec from UTIs	138	109 (79.0%)	10.3 ± 2.3	29 (21.0%)	5.5 ± 2.6	<0.0001
Aggregated totals and averages	1757	1207 (68.7%)	10.3 ± 3.0	550 (31.3%)	4.8 ± 2.5	<0.0001

## Data Availability

All sequences were deposited in NCBI under the Bioproject accession Nos. PRJNA861272 and PRJNA1168579.

## Supplementary Materials

The following supporting information can be downloaded at https://www.mdpi.com/article/10.3390/antibiotics15050427/s1. Table S1: Prevalence of AMR genes for Healthy children; Table S2: Prevalence of AMR genes for Domestic Animals; Table S3: Prevalence of AMR genes for Urinary Tract Infection (UTI) patients.
