# Supplementary material for: Antimicrobial Resistance Gene Profiles in Integron-Positive and Integron-Negative Third-Generation Cephalosporin-Resistant E. coli from Human and Animal Sources
Source: Antibiotics (Basel). 2026 Apr 24;15(5):427. doi: 10.3390/antibiotics15050427 (PMC13203075; doi:10.3390/antibiotics15050427)
Supplement: Supplementary file 1 [file antibiotics-15-00427-s001.zip › antibiotics-4169448-supplementary.pdf]

Supplementary Materials

Supplemental Table S1: Prevalence of AMR genes for Healthy children

	AMR_GROUP	integronState	numIsoCarrier	prevalence
1	Aminoglycoside	negative	92	9.725159
2	Aminoglycoside	positive	609	64.376321
3	Beta-lactam	negative	289	30.549683
4	Beta-lactam	positive	614	64.904863
5	Fosfomycin	negative	103	10.887949
6	Fosfomycin	positive	249	26.321353
7	Lincosamides	positive	13	1.374207
8	Macrolide	negative	15	1.585624
9	Macrolide	positive	174	18.393235
10	Multidrug	negative	300	31.712474
11	Multidrug	positive	621	65.644820
12	Phenicol	negative	32	3.382664
13	Phenicol	positive	339	35.835095
14	Polymyxin	negative	12	1.268499
15	Polymyxin	positive	11	1.162791
16	Quinolone	negative	116	12.262156
17	Quinolone	positive	180	19.027484
18	Rifamycin	positive	4	0.422833
19	Sulfonamide	negative	85	8.985201
20	Sulfonamide	positive	580	61.310782
21	Tetracycline	negative	137	14.482030
22	Tetracycline	positive	484	51.162791
23	Trimethoprim	negative	28	2.959831
24	Trimethoprim	positive	524	55.391121

Supplemental Table S2: Prevalence of AMR genes for Domestic Animals

	AMR_GROUP	integronState	numIsoCarrier	prevalence
1	Aminoglycoside	negative	89	13.2243685
2	Aminoglycoside	positive	440	65.3789004
3	Beta-lactam	negative	176	26.1515602
4	Beta-lactam	positive	455	67.6077266
5	Fosfomycin	negative	73	10.8469539
6	Fosfomycin	positive	256	38.0386330
7	Lincosamides	positive	9	1.3372957
8	Macrolide	negative	3	0.4457652
9	Macrolide	positive	80	11.8870728
10	Multidrug	negative	205	30.4606241
11	Multidrug	positive	460	68.3506686
12	Phenicol	negative	45	6.6864785
13	Phenicol	positive	347	51.5601783
14	Polymyxin	negative	7	1.0401189
15	Polymyxin	positive	20	2.9717682
16	Quinolone	negative	74	10.9955423
17	Quinolone	positive	159	23.6255572
18	Sulfonamide	negative	63	9.3610698
19	Sulfonamide	positive	430	63.8930163
20	Tetracycline	negative	115	17.0876672
21	Tetracycline	positive	404	60.0297177
22	Trimethoprim	negative	19	2.8231798
23	Trimethoprim	positive	393	58.3952452

Supplemental Table S3: Prevalence of AMR genes for Urinary Tract Infection (UTI) patients.

	AMR_GROUP	integronState	numIsoCarrier	prevalence
1	Aminoglycoside	negative	20	14.4927536
2	Aminoglycoside	positive	107	77.5362319
3	Beta-lactam	negative	25	18.1159420
4	Beta-lactam	positive	109	78.9855072
5	Fosfomycin	negative	7	5.0724638
6	Fosfomycin	positive	20	14.4927536
7	Macrolide	negative	2	1.4492754
8	Macrolide	positive	87	63.0434783
9	Multidrug	negative	29	21.0144928
10	Multidrug	positive	109	78.9855072
11	Phenicol	negative	2	1.4492754
12	Phenicol	positive	22	15.9420290
13	Quinolone	negative	2	1.4492754
14	Quinolone	positive	7	5.0724638
15	Rifamycin	positive	3	2.1739130
16	Sulfonamide	negative	9	6.5217391
17	Sulfonamide	positive	103	74.6376812
18	Tetracycline	negative	15	10.8695652
19	Tetracycline	positive	90	65.2173913
20	Trimethoprim	negative	1	0.7246377
21	Trimethoprim	positive	106	76.8115942
